# AT-BSS: A Broker Selection Strategy for Efficient Cross-Shard Processing in Sharded IoT–Blockchain Systems

**DOI:** 10.3390/s26082296

**Published:** 2026-04-08

**Authors:** Yue Su, Yang Xiang, Kien Nguyen, Hiroo Sekiya

**Affiliations:** 1 Graduate School of Science and Engineering, Chiba University, Chiba 263-8522, Japan; suyue@chiba-u.jp (Y.S.); 22wd5201@student.gs.chiba-u.jp (Y.X.); sekiya@faculty.chiba-u.jp (H.S.); 2 Institute for Advanced Academic Research, Chiba University, Chiba 263-8522, Japan

**Keywords:** IoT–blockchain, blockchain sharding, Broker mechanism, high performance blockchain system

## Abstract

The deep integration of the Internet of Things (IoT) and blockchain technology enables emerging applications in multi-party collaboration and trusted data sharing. However, the scalability constraints of blockchain networks remain a major bottleneck when handling high-frequency interactions in IoT–blockchain systems. Sharding addresses this challenge by partitioning the blockchain network into parallel sub-networks. Nevertheless, it introduces significant coordination overhead for cross-shard transactions. Among mitigation strategies, Broker-based mechanisms (e.g., BrokerChain) have attracted increasing attention for their efficiency in handling cross-shard communication by reducing verification overhead and communication latency. Despite these advantages, existing research typically treats the Broker group as a fixed configuration, neglecting the impact of Broker selection on system performance. To bridge this gap, this paper proposes the Accumulative Activity–Temporal Liveness Broker Selection Strategy (**AT-BSS**) to optimize cross-shard transaction processing in sharded IoT–blockchains. Specifically, we formally characterize the *Accumulative Activity* and *Temporal Liveness* of accounts in the account–transaction network and use these two metrics to identify accounts that maximize transaction-aggregation efficiency. We implement AT-BSS on the *BlockEmulator* platform and evaluate it against two baselines, namely, ABChain and BrokerChain. Under different settings of the number of Brokers (BrokerNum), number of shards (ShardNum), transaction arrival rate (InjectSpeed), and maximum block size (MaxBlockSize), AT-BSS consistently outperforms both baselines in terms of Transactions Per Second (TPS), Transaction Confirmation Latency (TCL), and Cross-shard Transaction Ratio (CTX). Compared with ABChain, AT-BSS achieves up to 15.5% higher TPS and reduces TCL and CTX by up to 80.2% and 28.7%, respectively. AT-BSS yields more pronounced results over BrokerChain, with TPS improvements of up to 229% and reductions of up to 97.7% in TCL and 80.5% in CTX.

## 1. Introduction

The rapid development of the Internet of Things (IoT) has led to a significant increase in the scale and heterogeneity of connected devices. Therefore, traditional centralized management models face growing limitations in data sharing efficiency, cross-entity collaboration, and system reliability. Blockchain offers a promising solution to these challenges through its decentralized architecture, data immutability, and traceability. By providing a robust and trustworthy foundation, it enables a new paradigm often referred to as IoT–blockchain [1,2]. However, the limited scalability [3,4] of blockchain systems remains a critical barrier to their large-scale adoption in IoT applications. For instance, Bitcoin supports only about seven transactions per second, with confirmation delays of up to 10 min [5]. Similarly, the average throughput of the Ethereum mainnet is only around 30 TPS [6]. Such throughput and latency levels are insufficient to meet the strict requirements of IoT services, which demand high concurrency and low latency.

To overcome scalability bottlenecks, sharding technology is widely regarded as an effective solution for balancing system throughput and decentralization [7,8,9]. This approach partitions the blockchain network into multiple shards that process transactions concurrently, thereby improving on-chain processing capacity while preserving decentralization and security. Among existing sharding protocols, BrokerChain [10] is notable for its efficient cross-shard processing capabilities. Compared with earlier approaches such as Monoxide’s Relay mechanism [11], BrokerChain introduces Broker accounts to restructure the transaction workflow. Instead of relying on complex cross-shard communication and verification, cross-shard transactions are decomposed into intra-shard transactions via Brokers. This effectively reduces the overhead associated with cross-shard synchronization and message interaction, which improves overall system efficiency.

Although the number of Brokers (BrokerNum) has been widely studied for its impact on system performance, far less attention has been paid to how Broker accounts should be selected. Current work on BrokerChain and its derivatives mostly relies on predefined Broker lists and lacks exploration of the correlation between Broker account attributes and system performance. Furthermore, existing studies lack a formal framework for modeling and analyzing account features. Therefore, systems operating under default configurations struggle to explore and achieve potential optimal performance ranges, which can unnecessarily limit scalability and cross-shard processing efficiency. Although ABChain [12] proposes a dynamic Broker selection mechanism, its optimization is still limited to adjusting the number and combination of Brokers within a fixed candidate list. While this candidate list is derived from high-frequency accounts among the first few transactions in the dataset, this approach lacks systematic justification for the list’s construction and representativeness. It also fails to establish a formalized model of account behavioral characteristics and does not analyze how the composition of the candidate Broker list impacts overall system performance.

To bridge this gap, this paper proposes the Accumulative Activity–Temporal Liveness Broker Selection Strategy (**AT-BSS**). Our contributions are summarized as follows:

- To the best of our knowledge, this study is the first to explicitly identify Broker account selection as a decisive factor affecting the performance of Broker-based sharded blockchains. Unlike prior work, which has focused primarily on tuning BrokerNum, we demonstrate that the attributes and distribution of Broker accounts also play a critical role. We elevate the Broker selection mechanism to a design problem that can be formally modeled and analyzed.
- We propose the Accumulative Activity and Temporal Liveness Broker Selection Strategy (AT-BSS). By capturing the heavy-tailed participation and bursty temporal patterns observed in historical transaction traces, we construct a quantifiable behavioral model for accounts. We formally define *Accumulative Activity* as an account’s aggregate transaction participation, and *Temporal Liveness* as the consistency of its participation over time. These metrics are integrated into a two-characteristic scoring function, providing an interpretable and computable framework for identifying optimal Broker candidates.
- We implement AT-BSS on the *BlockEmulator* platform and evaluate it against two state-of-the-art baselines, ABChain and BrokerChain. The evaluation results show that AT-BSS consistently outperforms both baselines. Specifically, it achieves higher Transactions Per Second (TPS), lower Transaction Confirmation Latency (TCL), and lower Cross-shard Transaction Ratio (CTX) across different settings of BrokerNum, ShardNum, InjectSpeed, and MaxBlockSize. In detail, relative to ABChain, AT-BSS improves TPS by up to 15.5% while cutting TCL and CTX by up to 80.2% and 28.7%. Relative to BrokerChain, the improvements are larger, reaching up to 229% in TPS and up to 97.7% and 80.5% reductions in TCL and CTX, respectively. These results highlight the crucial role of the Broker selection in cross-shard scheduling within sharded blockchain systems. Its proper design has a decisive impact on alleviating cross-shard bottlenecks and improving overall system scalability. It also provides a new perspective on system optimization in sharded blockchain systems.

The rest of this paper is structured as follows. Section 2 presents the background and related work. In Section 3, we analyze and verify the account characteristics. In Section 4, we introduce our proposal and the Broker-based sharding IoT–blockchain system with a Broker selection component in detail. Section 5 presents the experimental evaluation and results. Finally, we conclude this work in Section 6.

## 2. Background and Related Work

### 2.1. IoT–Blockchain Sharding Background

Sharding has established itself as the premier solution for addressing the scalability trilemma in high-concurrency IoT environments. By horizontally partitioning a network, sharding protocols enable parallel transaction processing, thereby reducing latency and increasing throughput. Cai X et al. [13] address shard failure in industrial IoT–blockchains caused by the random aggregation of malicious nodes. They first formulate shard failure as a closed-form binomial probability. This probability is then incorporated into a four-objective Shard-Validation-Validity Model (SVVM) that also considers throughput, delay, and malicious-node load. Li M et al. [14] propose NsScheme, a static scheme designed for lightweight IoT–blockchain access control in a cloud–edge–device architecture. NsScheme is the first static scheme that simultaneously considers the two stages of network sharding and transaction allocation. Cai T et al. [15] point out that static sharding is difficult to sustain in mobile IoT–blockchains. Frequent device joining and leaving, heterogeneous computing power, and base station switching make it hard to maintain the security–throughput trade-off over time. To address this issue, the authors formulate the sharding reconstruction problem as an MDP and define three quantifiable performance metrics: consensus latency, reconstruction latency, and security penalty. Based on this formulation, they propose the SmartChain framework.

**Transaction model:** Blockchain transaction models primarily fall into two categories: the Unspent Transaction Output (UTXO) model [16] and the Account/Balance model [17]. The UTXO model is the basis of systems such as Bitcoin, where each transaction consumes existing unspent outputs and generates new ones, analogous to making change in cash transactions. This model offers advantages in decentralized verification, privacy, and security, but because the state is expressed and updated in discrete UTXOs, it can be inefficient in smart contract scenarios with frequent state changes and complex logic. In contrast, the Account/Balance model is widely used in smart contract platforms such as Ethereum. It records asset ownership and system state through account balances and contract states. As a result, it is well suited for applications with frequent state updates and supports programmable logic and complex interactions.

**Broker mechanism:** To handle cross-shard transactions [7,18], various mechanisms have been proposed under the Account/Balance model, such as the Relay mechanism and the Broker mechanism. Compared with the Relay mechanism, the Broker mechanism not only enables more efficient handling of cross-shard transactions but also decreases their proportion. By segmenting and distributing the state of selected Broker accounts across shards, Brokers can serve as intermediaries in cross-shard interactions. This design allows many cross-shard transactions to be transformed into intra-shard transactions, thus effectively lowering the rate of cross-shard transactions.

The Broker mechanism can be illustrated with the following example: Consider a cross-shard transaction in which account A in one shard transfers assets to account B in another shard. In the Broker mechanism, this transaction is divided into two intra-shard transactions. The first transaction occurs within the source shard, between account A and Broker account C, while the second occurs within the target shard, between account B and the same Broker account C. By decomposing a cross-shard transaction into two intra-shard transactions, this approach reduces coordination overhead and improves processing efficiency.

### 2.2. Related Work

As a representative approach to improving scalability, sharded blockchains have been extensively studied. Related work can be broadly categorized into two types: overall system performance improvement and cross-shard transaction optimization. For example, MVCom [19] proposes a committee scheduling strategy for large-scale sharded networks. It improves scalability by reducing the performance loss caused by committee formation and consensus latency. IShard [20] proposes a sharded consensus design for consortium blockchains that improves scalability and security and supports atomic processing of both intra-shard and cross-shard transactions. GradingShard [21] combines network sharding and transaction sharding. It uses VRF_POS-based node assignment and sender-address-based transaction partitioning to enable parallel transaction processing, thereby improving overall system throughput. The other category focuses on reducing the confirmation and coordination overhead introduced by cross-shard transactions. For instance, OptChain [22] targets a UTXO-based cryptocurrency ledger and uses smart transaction placement to aggregate related transactions into the same shard as much as possible, thereby reducing cross-shard transactions. Estuary [23] is designed as a general-purpose ledger and redesigns the state model through multi-level state splitting and aggregation as well as COPRAS-based state distribution. This design keeps most user-to-user transactions intra-shard and significantly reduces cross-shard operations. ControlShard [24], which is tailored for consortium blockchains, introduces a dedicated control shard to coordinate and confirm cross-shard transactions, thereby improving cross-shard processing efficiency.

Recent studies have also optimized cross-shard transactions through scheduling and placement, as well as load balancing. For example, Ren et al. [25] and Król et al. [26] reduce cross-shard overhead through transaction/object placement and migration. Zhang et al. [27] further leverage historical transaction patterns to jointly optimize cross-shard transaction ratio and shard workload balance. From the perspective of hot-account mitigation, Li et al. [28] improve sharding performance through account migration. Nevertheless, these approaches mainly optimize cross-shard efficiency through placement, migration, or load balancing, rather than through Broker design.

Inspired by the Broker mechanism in [10], a series of follow-up schemes have been proposed. For instance, Zheng et al. [29] build a DeFi dApp where users can become Brokers by staking tokens and providing liquidity, earning rewards while improving cross-shard transaction efficiency. Chen et al. [30] adopt a similar incentive-driven design to encourage user participation as Brokers, aiming to increase system liquidity and shorten cross-shard confirmation latency. We summarize these related studies in Table 1.

Our proposal is motivated by empirical findings from prior studies of large-scale historical Ethereum transactions [31,32,33,34,35,36,37]. These studies consistently indicate two important account-level characteristics: (1) transaction participation is highly skewed, with a small number of accounts participating in a large volume of transactions, and (2) account activity exhibits strong temporal burstiness, characterized by short intervals of intense transactions interleaved with prolonged inactivity. Despite these observations, existing studies have mainly described transaction network structures and behavioral regularities, without leveraging these findings for principled Broker account selection in sharded blockchain systems.

## 3. Account Analysis in Account–Transaction Network

To provide an empirical basis for the design of our Broker selection strategy, this section analyzes account behaviors in historical Ethereum transaction networks. Consistent with established methodologies in Broker-based sharding research (e.g., ABChain [12]), we utilize Ethereum historical transaction data for account-based IoT–blockchain environments. We focus on two key characteristics: Accumulative Activity, which measures the intensity of an account’s participation as a sender/receiver in the account–transaction network, and Temporal Liveness, which measures the account’s sustained activity over time.

### 3.1. Accumulative Activity Characteristic

#### 3.1.1. Definition of Equation

We model the Ethereum historical transaction data as a weighted directed account–transaction network G=(V,E,W) to characterize both the interaction intensity among accounts and their transaction participation scale. Here, *V* is the set of vertices, *E* is the set of directed edges, and *W* assigns a weight to each edge. In detail, each vertex v∈V corresponds to an account address in the account–transaction network. For any two accounts u,v∈V, we add a directed edge (u→v)∈E if there exists at least one transaction whose sender is *u* and receiver is *v*. The edge weight $w_{uv}\in W$ denotes the number of transactions occurring along the direction u→v. Specifically,

(1) $$w_{uv}=\left|\left\{\;t\in T\;|\;\mathrm{src}(t)=u,\;\mathrm{dst}(t)=v\;\right\}\right|.$$

where T denotes the set of transactions in the account–transaction network. src(·) and dst(·) indicate the sender and receiver of a transaction, respectively.

Based on *G*, we quantify an account’s *Accumulative Activity* as its total transaction participation volume, accounting for both sender and receiver. Specifically, the overall number of transactions is

(2) $$N_{\mathrm{total}}\left(a\right)=N_{\mathrm{out}}\left(a\right)+N_{\mathrm{in}}\left(a\right).$$

where $N_{\mathrm{out}}\left(a\right)$ and $N_{\mathrm{in}}\left(a\right)$ denote the outgoing and incoming accumulative transaction counts of account *a*, respectively, given by

(3) $$N_{\mathrm{out}}\left(a\right)=\sum_{v\in V}w_{av},\;N_{\mathrm{in}}\left(a\right)=\sum_{u\in V}w_{ua}.$$

where $w_{av}$ denotes the number of transactions sent from account *a* to account *v*, while $w_{ua}$ denotes the number of transactions sent from account *u* to account *a* (i.e., the edge weights on a→v and u→a, respectively).

#### 3.1.2. Results of Analysis

The transaction data used in this study is drawn from the Ethereum on-chain dataset released by XBlock [38]. It collects Ethereum on-chain data, including blocks, transaction traces, and receipts, by running full nodes. It then organizes the data into multiple sub-datasets, such as Block, BlockTransaction, and ContractInfo. We use the BlockTransaction dataset for analysis. The dataset is divided into more than 20 files, from which we select one file for analysis. The data mainly consists of detailed block information related to the transfer transaction, including fields such as sender address, receiver address, and transfer amount. More detailed information is shown in Table 2. Although the Ethereum historical transaction dataset provides a large-scale and realistic account-based dataset for analyzing account behavior, it does not cover all IoT–blockchain workloads. We choose Ethereum because it is publicly available, comes from a real transaction network, and is suitable for observing account behavioral characteristics. Ethereum accounts are not simply treated as direct equivalents of a specific type of entity in IoT environments. Instead, they are regarded as participating entities in an account-based transaction network. In IoT–blockchain systems, such entities may correspond to various types of participants depending on the system design. These include device nodes, gateway nodes, edge service nodes, and other entities engaged in data interaction and transaction recording. Their practical roles and application semantics are different from those of Ethereum accounts. Even so, they may still exhibit similar behavioral characteristics at the transaction-network level, such as participation frequency, sustained activity, and interaction concentration. However, IoT-oriented systems differ from Ethereum in transaction value, device communication regularity, temporal burstiness, and entity locality. Therefore, Ethereum is treated here as a starting point for studying behavior-driven Broker selection in account-based transaction networks rather than as a complete representation of all IoT deployment scenarios. Evaluating AT-BSS on IoT-native workloads remains important future work.

To analyze account Accumulative Activity in different account–transaction networks, we randomly select two transaction data files from different parts of the BlockTransaction subset. We then construct the corresponding account–transaction networks and analyze their distributions. This helps reduce random bias from a single time interval or a single account–transaction network and allows us to verify whether the observed characteristics of account Accumulative Activity are consistent across datasets. We compute $N_{\mathrm{total}}\left(a\right)$ for all accounts and visualize them in two datasets. Dataset 1 corresponds to BlockTransaction 2,000,000–2,999,999, and Dataset 2 corresponds to BlockTransaction 3,000,000–3,999,999. More detailed information about the time span and the number of accounts for both datasets is displayed in Table 3.

**Figure 1:**
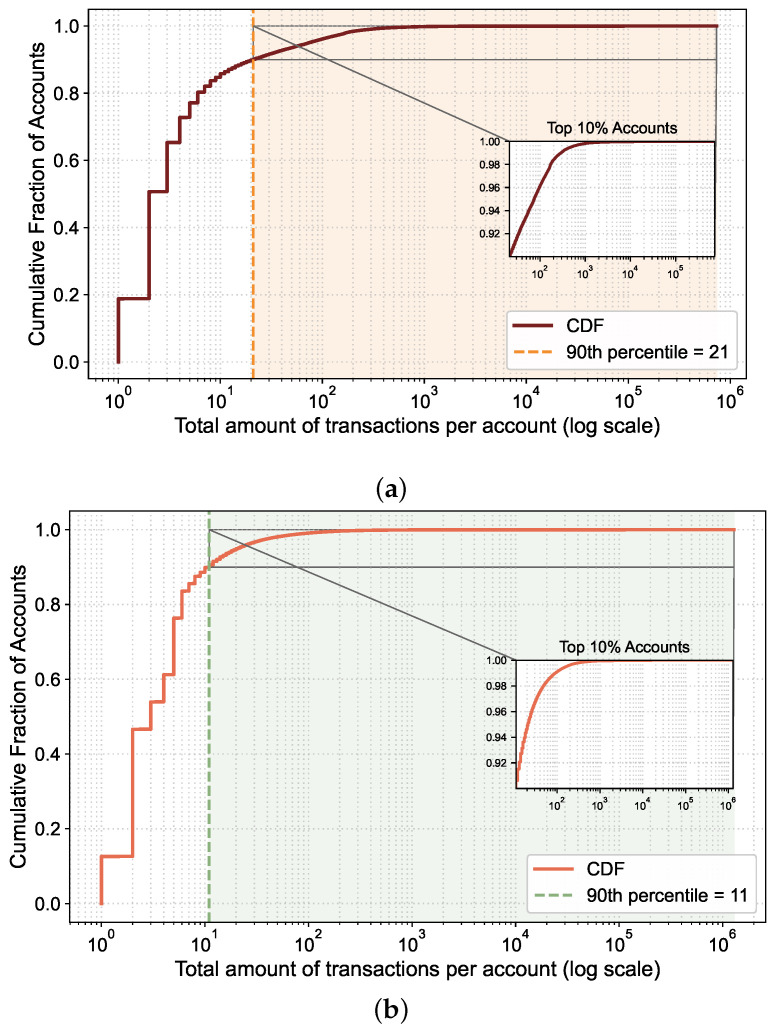
CDF of accounts’ Accumulative Activity in (**a**) Dataset 1 and (**b**) Dataset 2.

The results of the account characteristic analysis are shown in Figure 1a,b, which plot the CDF of the total number of transactions per account. Both results exhibit a highly consistent skewed pattern. More specifically, the CDF increases sharply in the low-activity range, which indicates that most accounts are involved in only a few transactions. Meanwhile, the long tail on the right side of the logarithmic x-axis suggests that a small number of accounts participate far more actively than the average. The inset further shows that even within the top 10% most active accounts, Accumulative Activity still differs by orders of magnitude, indicating a clear stratification among the top accounts. Although both results exhibit an overall heavy-tailed distribution, the activity levels of accounts differ. In Dataset 1, the 90th percentile threshold is 21, meaning that approximately 90% of accounts have a cumulative transaction participation of no more than 21. In Dataset 2, this threshold drops to 11, indicating that the majority of accounts have sparser transaction participation within the latter block interval.

### 3.2. Temporal Liveness Characteristic

#### 3.2.1. Definition of Equation

To characterize the activity patterns of accounts in the account–transaction network over time, we map the timestamp of each transaction in the dataset to a daily granularity in UTC. Then, we include both the sender and receiver accounts for each transaction in the statistics. If an account appears at least once as either a sender or receiver on a given day, it is considered active on that day. Based on this, we construct a binary account–date matrix. Let $D=\{d_{1},d_{2},\ldots ,d_{T}\}$ be the set of days within the observation window, and |D| is the total number of days. For any account a∈V and any day $d_{t}\in D$, we define a binary activity indicator

(4) $$L\left(a,d_{t}\right)=\left\{\begin{array}{cc} 1, & \mathrm{if}\;\mathrm{account}\;a\;\mathrm{appears}\;\mathrm{in}\;\mathrm{at}\;\mathrm{least}\;\mathrm{one}\;\mathrm{transaction}\;\mathrm{on}\;\mathrm{day}\;d_{t},\\ 0, & \mathrm{otherwise}. \end{array}\right.$$

Accordingly, we construct a binary matrix L capturing the liveness of all accounts over the entire time window. Its entry is defined as $L_{i,t}=L\left(a_{i},d_{t}\right)$, where $a_{i}$ denotes the *i*-th account (i=1,…,|V|) and $d_{t}$ denotes the *t*-th day in the observation window (t=1,…,|D|). By construction, $L_{i,t}\in \left\{0,1\right\}$ and L captures only whether an account is present on a given day regardless of how many transactions occur on that day. This representation highlights accounts’ temporal appearance patterns (e.g., continuously active, intermittently active, or long-term dormant), thereby reflecting their temporal liveness characteristics.

#### 3.2.2. Results of Analysis

**Figure 2:**
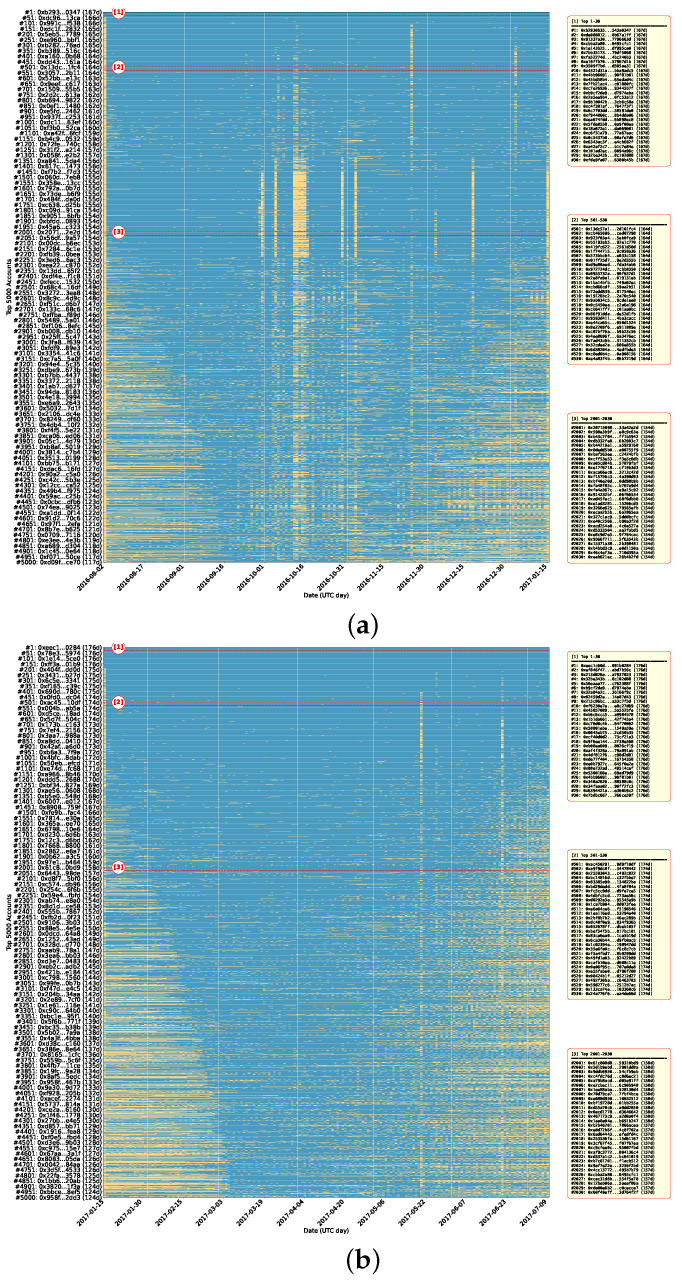
Temporal liveness maps for the top-5000 accounts in (**a**) Dataset 1 and (**b**) Dataset 2, ordered by active-day count (the x-axis represents date and the y-axis represents account rank. Blue denotes that the account is active on that day, whereas yellow indicates that the account is inactive).

We visualize Dataset 1 and Dataset 2 in Figure 2. Due to the massive number of accounts in both datasets, we select the top 5000 accounts with the most active days for analysis in each dataset. In the figure, the horizontal axis shows the daily timeline, and the vertical axis shows the top 5000 accounts ranked by active days, with account addresses as labels. To avoid visual clutter, we display account addresses on the y-axis at 50-account intervals rather than labeling each account. In addition, the zoom-in boxes on the right highlight several representative rank ranges, including top 1–30, 501–530, and 2001–2030. For each range, they list the corresponding account addresses and active-day counts. Higher-ranking accounts have participated in transactions over more days. Each row corresponds to one account. A blue bar on a given date indicates that the account is active (i.e., involved in at least one transaction) on that day, whereas yellow indicates inactivity with no transaction records.

As shown in Figure 2a, Dataset 1 covers the period from 2 August 2016 to 15 January 2017 (approximately 5–6 months). The results show that top-ranked accounts have high activity coverage over time. For example, the top-one account had 167 active days within the time window, indicating that high-ranking accounts such as this are not momentarily active entities but rather capable of sustained transactions over a relatively long period. In contrast, as account rankings decline, the number of active days for most accounts decreases significantly. The activity patterns gradually shift from relatively continuous to scattered and sparse. Furthermore, the figure shows several clear vertical bands, where many accounts become active on the same dates. Many accounts show concentrated activity on a few specific dates, forming dense vertical bands. This pattern indicates temporal burstiness in account participation. The results for Dataset 2 are shown in Figure 2b. The time window covers the period from 15 January 2017 to 9 July 2017 (approximately 6 months). Similarly, a few of the top-ranked accounts maintain a relatively consistent activity trajectory, with the top-one account reaching 176 active days. However, as account rankings decline, the distribution of activity bands on the timeline gradually shifts from dense to more intermittent and sparse, reflecting a significant difference between a few highly active accounts and a large number of inactive accounts. Similarly, Dataset 2 also shows clear date-specific activity bands, in which many accounts are active on certain dates, while activity remains sparse on others.

## 4. Accumulative Activity–Temporal Liveness Broker Selection Strategy (AT-BSS)

The Broker mechanism’s effectiveness depends heavily on Broker selection. If chosen Brokers have few transactional ties to most ordinary accounts or remain inactive for long periods, few transactions will pass through them, so cross-shard verification still occurs and overhead barely drops. In contrast, Brokers that interact frequently with many accounts and stay active are more likely to keep transactions within a single shard, reducing cross-shard messages and improving performance. Different Broker sets therefore reshape cross-shard interaction patterns and system efficiency.

Based on the above motivations, we propose a novel Broker account selection strategy. The key idea is to replace a random/preset Broker list with a data-driven, measurable selection process. The design of this strategy follows two principles: (1) Brokers should maintain dense and persistent transactional links with many ordinary accounts. This allows more transactions to be routed through Brokers and reduces direct cross-shard interactions. (2) Brokers should remain continuously or frequently active over time to ensure stable relay capacity and avoid performance degradation caused by prolonged inactivity. Guided by these two principles and our analysis of account behavior in the account–transaction network, the strategy is built on two characteristics: Accumulative Activity and Temporal Liveness. Accumulative Activity captures the long-term participation intensity of an account in the transaction network, reflecting its potential to aggregate and relay cross-shard transactions. Temporal Liveness captures the continuity and regularity of account activity over time, which is important because highly active accounts that are only bursty or short-lived may not be reliable Broker candidates. Therefore, these two metrics are intended to represent two complementary and vital aspects of Broker suitability: participation scale and sustained temporal activeness. This strategy is therefore referred to as AT-BSS. In AT-BSS, we fuse these two characteristics into a comprehensive score for each account to quantify its effectiveness as a Broker. AT-BSS calculates a score for each ordinary account, sorts them from highest to lowest score, and selects the top-*K* accounts to form a Broker list, where *K* is the number of Brokers configured in the system.

Unlike BrokerChain and ABChain, AT-BSS introduces two core aspects of Broker selection. First, instead of relying on a built-in predefined Broker list, AT-BSS constructs the Broker set by ranking ordinary accounts with an account–transaction network-driven scoring function at the account level. Second, AT-BSS explicitly incorporates both Accumulative Activity and Temporal Liveness into the scoring principle, so that Broker selection depends not only on participation intensity but also on sustained activity over time. Therefore, the novelty of AT-BSS lies not merely in changing the Broker list itself but more importantly in formalizing Broker selection as a measurable account-behavior modeling problem. To further clarify the differences between AT-BSS and baselines, Table 4 provides a concise side-by-side comparison.

Moreover, the Broker list in AT-BSS is determined at the start of each system run and remains fixed throughout execution. Specifically, for a given BrokerNum setting, AT-BSS selects the top-*K* Broker accounts based on its ranking criteria before transaction processing starts. The resulting Broker list then remains unchanged throughout that run. The Broker list is reconstructed only when BrokerNum changes. Therefore, for a fixed BrokerNum, AT-BSS does not perform Broker list updates, periodic reselection, or rolling online replacement. As a result, there is no update frequency or overhead associated with AT-BSS.

### 4.1. Notation for AT-BSS

Let T be the transaction set and A be the account set. For each transaction e∈T, $t_{e}$ denotes its timestamp and V(e)⊆A denotes the set of participating accounts (including the sender and receiver).

### 4.2. Quantify Accumulative Activity

We define an account-level participation indicator as

(5) $$I_{a}\left(e\right)=\left\{\begin{array}{cc} 1, & a\in V(e),\\ 0, & \mathrm{otherwise}. \end{array}\right.$$

where $I_{a}\left(e\right)=1$ means that account *a* appears in transaction *e* as either the sender or the receiver. Otherwise, $I_{a}\left(e\right)=0$. This binary function converts per-transaction involvement into a countable quantity.

The number of transactions participated in by account *a* is

(6) $$N\left(a\right)=\sum_{e\in T}I_{a}\left(e\right).$$

A larger N(a) indicates that account *a* participates in more transactions and thus has a wider transactional reach in the account–transaction network. We apply a logarithmic compression and define the Accumulative Activity as

(7) $$A\left(a\right)=\ln\left(1+N(a)\right)=\ln\left[1+\sum_{e\in T}I_{a}\left(e\right)\right].$$

We use logarithmic compression because account participation in historical transaction networks typically follows a highly skewed long-tail distribution, where a small number of accounts have extremely large transaction counts. Using the raw participation count directly would make the score dominated by a small number of extremely active accounts. By applying a logarithmic transformation, this scale gap can be compressed while preserving the relative ordering of activity levels, making the metric more stable and suitable for ranking candidate Broker accounts.

### 4.3. Quantify Temporal Liveness

Let $E_{a}=\langle e_{1},e_{2},\ldots ,e_{N(a)}\rangle$ be the time-ordered sequence of transactions in which account *a* participates (i.e., $t_{e_{1}}<t_{e_{2}}<\ldots <t_{e_{N(a)}}$). $E_{a}$ is obtained by collecting all transactions involving account *a* and sorting them by timestamp. This ordering allows us to further measure how active account *a* is in the account–transaction network. More specifically, we define the mean inter-transaction interval of account *a* as

(8) $$\Delta t\left(a\right)=\frac{1}{N(a)-1}\sum_{i=2}^{N(a)}\left(t_{e_{i}}-t_{e_{i-1}}\right).$$

where Δt(a) averages the time gaps between two consecutive transactions in $E_{a}$. We choose the mean inter-transaction interval because the purpose of this metric is to capture whether an account remains continuously and frequently active over time. Compared with more complex temporal statistics, the mean interval provides a simple and intuitive measure of average activity continuity. A smaller Δt(a) means *a* participates more frequently, indicating stronger temporal activeness. We introduce a smoothing constant $\epsilon =10^{-9}$ and define the Temporal Liveness as

(9) $$T\left(a\right)=\ln\left[\frac{1}{\Delta t(a)+\epsilon }\right].$$

### 4.4. Total Score

Finally, we combine the two metrics into a unified score:

(10) Score(a)=A(a)+T(a).

Given the system-configured number of Brokers *K*, we select the top-*K* accounts with the highest Score(a) values as the Broker set. We adopt an additive combination for the score function for three reasons. First, it provides an interpretable and lightweight ranking rule that is easy to compute and deploy in systems. Second, the current study aims to use a simple and reproducible scoring method as a starting point before considering more complex weighting or learning-based strategies. Third, the equal-weight addition reflects that Accumulative Activity and Temporal Liveness are equally important complementary dimensions, and the two are independent of each other.

### 4.5. AT-BSS Algorithm

Algorithm 1 provides pseudocode for the complete procedure of AT-BSS, including steps such as data preprocessing, statistical aggregation, score calculation, sorting, and Broker list generation. In more detail, AT-BSS begins by sorting all transactions in T in ascending order of timestamps (Line 2). This ordering guarantees that the transaction occurrences associated with each account can be organized chronologically, which is necessary for subsequent temporal calculations. For each a∈A, the transaction participation amount is calculated by Equation (6) to quantify the overall participation scale (Line 4). Then the time-ordered transaction sequence is built and the mean inter-transaction interval is computed by Equation (8), which characterizes how frequently the account appears over time (Lines 5–6). After that, the Accumulative Activity and the Temporal Liveness are calculated by Equations (7) and (9). These two components are then fused into the combined score by Equation (10) (Lines 10–12). After scoring all accounts, the algorithm ranks them by sorting in descending order (Line 14). The top-*K* accounts in the ranking are selected to form the Broker list *B* (Line 16). Finally, the selected addresses are normalized to the required format (e.g., removing the “0x” prefix) and output as the Broker list.
**Algorithm 1** AT-BSS**Require:**  Transaction set T; Account set A; Number of Brokers *K*; Participant account set V(e) **Ensure:**  Broker list B   1:**// Step1: Data preprocessing & statistics:**  2:Sort transactions in T by timestamp (ascending order)  3:**for** each account a∈A **do**  4:   Compute transaction count N(a) by Equation (6)  5:   Build time-ordered sequence $E_{a}=\langle e_{1},\ldots ,e_{N(a)}\rangle$  6:   Calculate mean inter-transaction interval Δt(a) by Equation (8)  7:**end for**  8:**// Step2: Score calculation & ranking:**  9:**for** each account a∈A **do**10:   Compute A(a) by Equation (7)11:   Compute T(a) by Equation (9)12:   Compute Score(a) by Equation (10)13:**end for**14:Sort accounts by Score(a) in descending order15:**// Step3: Result output:**16:Select top-*K* accounts as B17:**return** B

### 4.6. Broker-Based Sharding IoT–Blockchain System with AT-BSS

**Figure 3:**
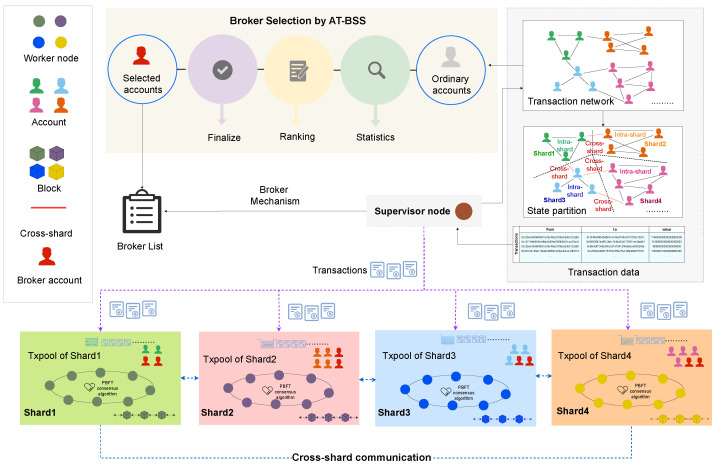
Implementation of Broker-based sharded IoT–blockchain system based on AT-BSS.

We implement the proposed AT-BSS within a sharded IoT–blockchain system on the *BlockEmulator* platform [10], as depicted in Figure 3. The IoT–blockchain network is partitioned into multiple shards. In each shard, participating nodes collaboratively execute transactions and generate blocks using the Practical Byzantine Fault Tolerance (PBFT) consensus algorithm. A leader node is elected in each shard to coordinate the intra-shard consensus workflow, including message exchange and transaction scheduling. The remaining nodes act as followers and are responsible for validating transactions, updating local states, and maintaining ledger consistency.

In addition, a Supervisor node is introduced to provide global coordination. It injects historical Ethereum transaction data, maintains the system state, handles shard management, and dispatches transactions to the transaction pool of each shard leader to trigger execution. Cross-shard interactions are realized via a Broker mechanism that enables collaborative processing and information relay among shards. To further improve cross-shard efficiency, we incorporate an AT-BSS module, which selects appropriate Broker accounts from ordinary accounts and deploys them in each shard as cross-shard intermediaries.

The system consists of two primary object types: nodes and accounts. Nodes are responsible for computation and consensus, whereas accounts initiate and receive transactions. Account-to-shard affiliation is deterministically derived from account identifiers via modulo operations. Specifically, for each account address, the last eight hexadecimal characters are extracted and converted into an unsigned integer. The resulting value is then taken modulo the total number of shards to determine the shard ID. See Equation (11) for the detailed formula. Therefore, the same account is always assigned to the same shard under a fixed shard-number setting. When the number of shards changes, the mapping is recalculated using the updated modulo base, and accounts are remapped accordingly. In addition, Broker accounts are selected globally from the Broker list rather than separately within each shard.

(11) $$\mathrm{ShardID}\left(a\right)=\mathrm{HexToUint}\;\left({\mathrm{suffix}}_{8}\left(a\right)\right)\;\mathrm{mod}\;\mathrm{ShardNum}$$

For communication, all nodes use the local loopback address (127.0.0.1) as a unified endpoint and are differentiated by distinct port numbers. This configuration represents a simplified, ideal network condition that reduces fluctuations caused by network factors. However, in real-world distributed deployments, factors such as network latency, jitter, and bandwidth constraints can affect the system’s overall throughput and latency, which limits the effectiveness of externalities. Inter-node interactions rely on TCP connections implemented using the Go standard library’s net package. In particular, tcpsock.go encapsulates core interfaces such as DialTCP and ListenTCP, providing stable and efficient support for connection establishment and data transmission among nodes.

### 4.7. Discussion

In the current study, Broker selection is based on historical Accumulative Activity and Temporal Liveness under a performance-oriented setting. The proposed AT-BSS does not introduce additional security assumptions beyond those already present in ABChain and BrokerChain, because it does not modify the underlying consensus mechanism or the cross-shard transaction validation procedure but only changes the Broker selection strategy. However, if some accounts behave strategically or maliciously, this design may introduce several security risks. Specifically, repeatedly selecting highly active and persistently active accounts may increase the concentration of Broker roles, leading to partial centralization and greater system reliance on a relatively small set of accounts. Moreover, because Broker selection depends on observable activity patterns, malicious accounts may attempt to inflate their activity statistics. Such manipulation could include fake transactions, transaction padding, or Sybil-style behavior aimed at increasing selection probability. Collusive behavior among selected accounts, or targeted inactivity of critical Brokers, may further affect the stability, robustness, and fairness of the selection outcome.

The present work focuses on the performance impact of Broker selection and does not explicitly incorporate anti-fraud or security-aware constraints into the AT-BSS rules. We therefore regard this as an important limitation for practical deployment. Possible mitigation directions include introducing diversity constraints or caps on repeated Broker assignments. Another option is to apply activity-quality filters to distinguish normal transactions from suspicious self-generated behavior. In addition, the current scoring mechanism can be extended with reputation, stake, or historical trust indicators. These mechanisms can be incorporated into the candidate filtering stage or the scoring stage of Broker selection. Such extensions would make AT-BSS more robust against manipulation, collusion, and inactivity-related attacks. We leave them to future work.

### 4.8. Complexity Analysis

The complexity of the AT-BSS algorithm can be analyzed in terms of time and space. Let M=|T| denote the number of transactions in the input transaction set, and let N=|A| denote the number of accounts.

AT-BSS first sorts the transaction set T by timestamp in ascending order, requiring O(MlogM) time. The subsequent per-account score computation requires O(N) time, as each score is evaluated exactly once. The accounts are then ranked in descending order, which requires O(NlogN) time. Therefore, the total time complexity of AT-BSS is O(MlogM+NlogN). For space complexity, storing the transaction set requires O(M) space. The account-level statistics require O(N) space. Thus, the overall space complexity of AT-BSS is O(M+N).

### 4.9. Practical Deployment Implications

From a practical deployment perspective, AT-BSS reduces runtime overhead because Broker scoring and ranking are completed before transaction processing begins rather than being repeated during system runtime. Furthermore, since the Broker set remains constant throughout the operation, it provides a stable execution environment, making the final system behavior easier to control and analyze. However, this design also involves trade-offs: while avoiding the cost of online reselection, it results in a slower response time to potential changes in account behavior during execution. Therefore, the current AT-BSS configuration is better suited to systems that prefer stable phased execution and low runtime overhead rather than continuous online adaptation.

## 5. Evaluation and Experimental Results

### 5.1. Experimental Settings

We implement and evaluate the proposed AT-BSS strategy using BlockEmulator. The experimental testbed is orchestrated on a PC (AMD Ryzen 7, 3.80 GHz, 24 GB RAM) running Windows 11. We select Dataset 1 for evaluation. We compare AT-BSS against two distinct baselines: BrokerChain and ABChain. For fair comparison, all methods are evaluated on the same dataset, with the same parameter settings, consensus configuration, and BlockEmulator environment. All three methods adopt the same default BrokerNum setting (BrokerNum = 10) unless otherwise specified. For the two baselines, the initial Broker candidate pool is a fixed list of 100 high-frequency accounts identified from the first 500 transactions in the dataset. While BrokerChain remains static throughout the runtime, ABChain updates its active Brokers by rotating them within this 100-account candidate list (i.e., the update interval is one epoch). In contrast, AT-BSS constructs its Broker list by ranking all candidates according to the proposed scoring function and selecting the top BrokerNum accounts. It is determined before each experimental run based on the configured BrokerNum and is not recomputed during a run. We first evaluate all three methods by varying only the Broker configuration. We set BrokerNum to 5, 10, 15, 20, 25, and 30 to evaluate system performance under different Broker settings. Next, with a fixed number of Brokers (i.e., BrokerNum = 10), we examine the impact of other key system parameters (i.e., ShardNum, InjectSpeed, MaxBlockSize) on system performance.

**Figure 4:**
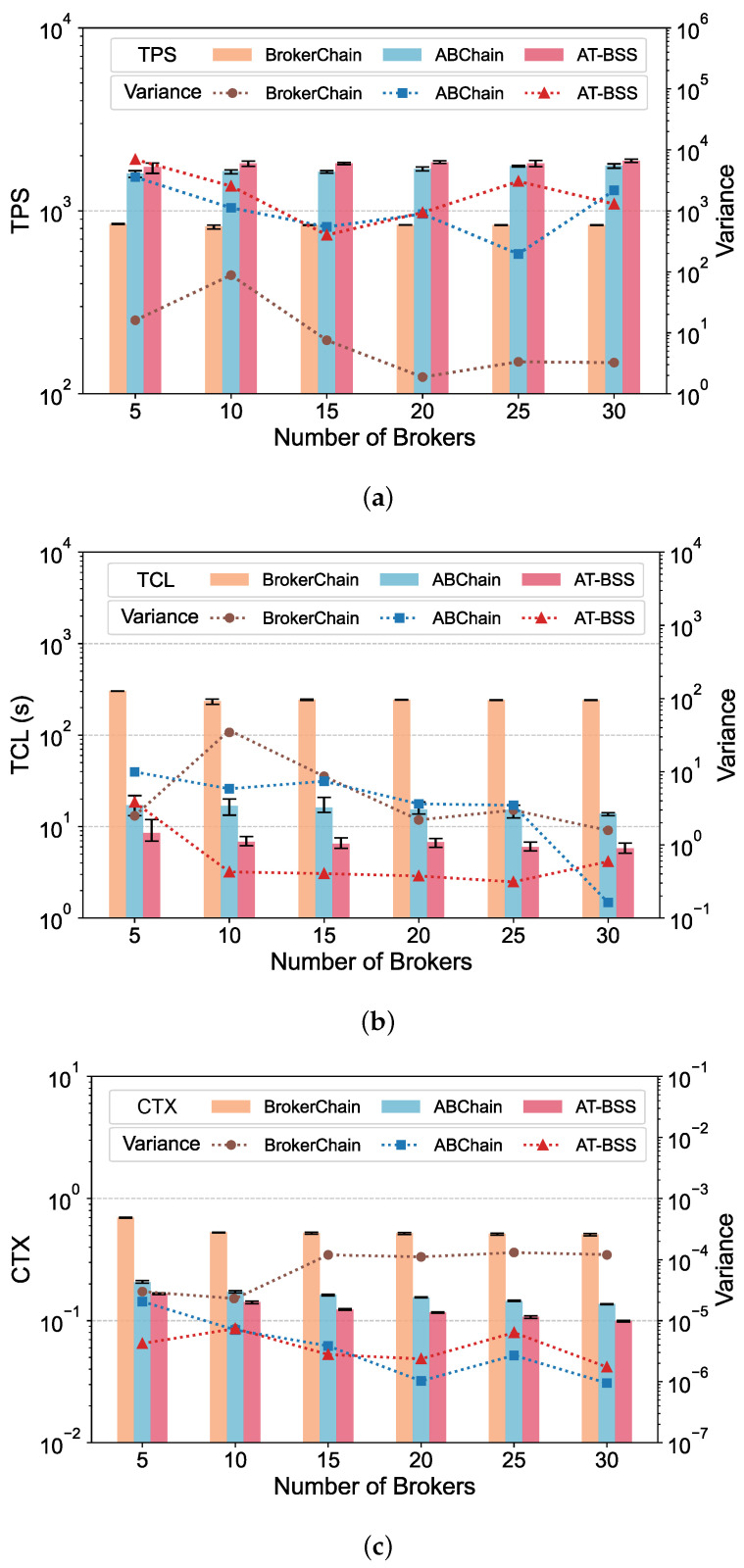
Performance comparison under varying Broker numbers: (**a**) TPS; (**b**) TCL; (**c**) CTX.

**Figure 5:**
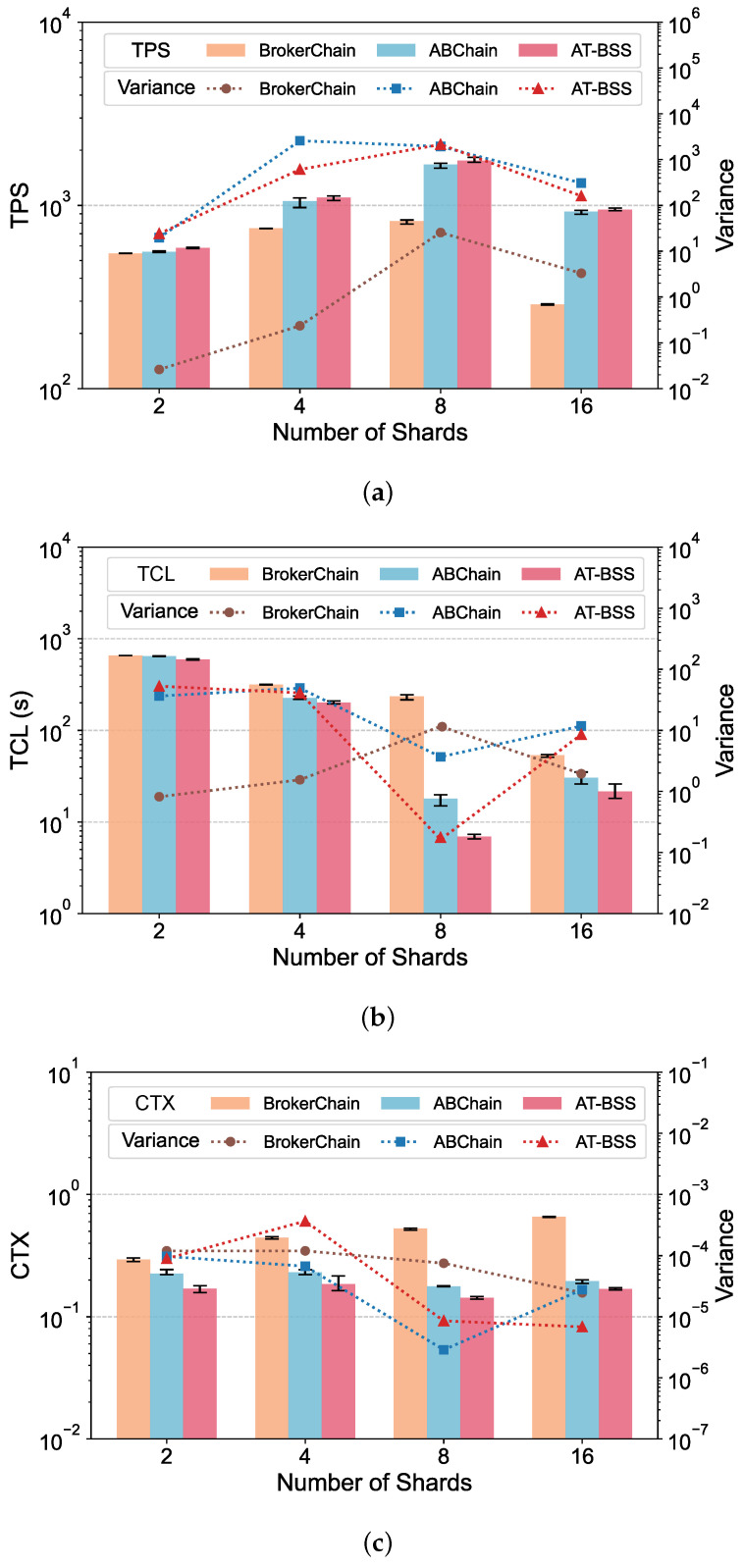
Performance comparison under varying shard numbers: (**a**) TPS; (**b**) TCL; (**c**) CTX.

**Figure 6:**
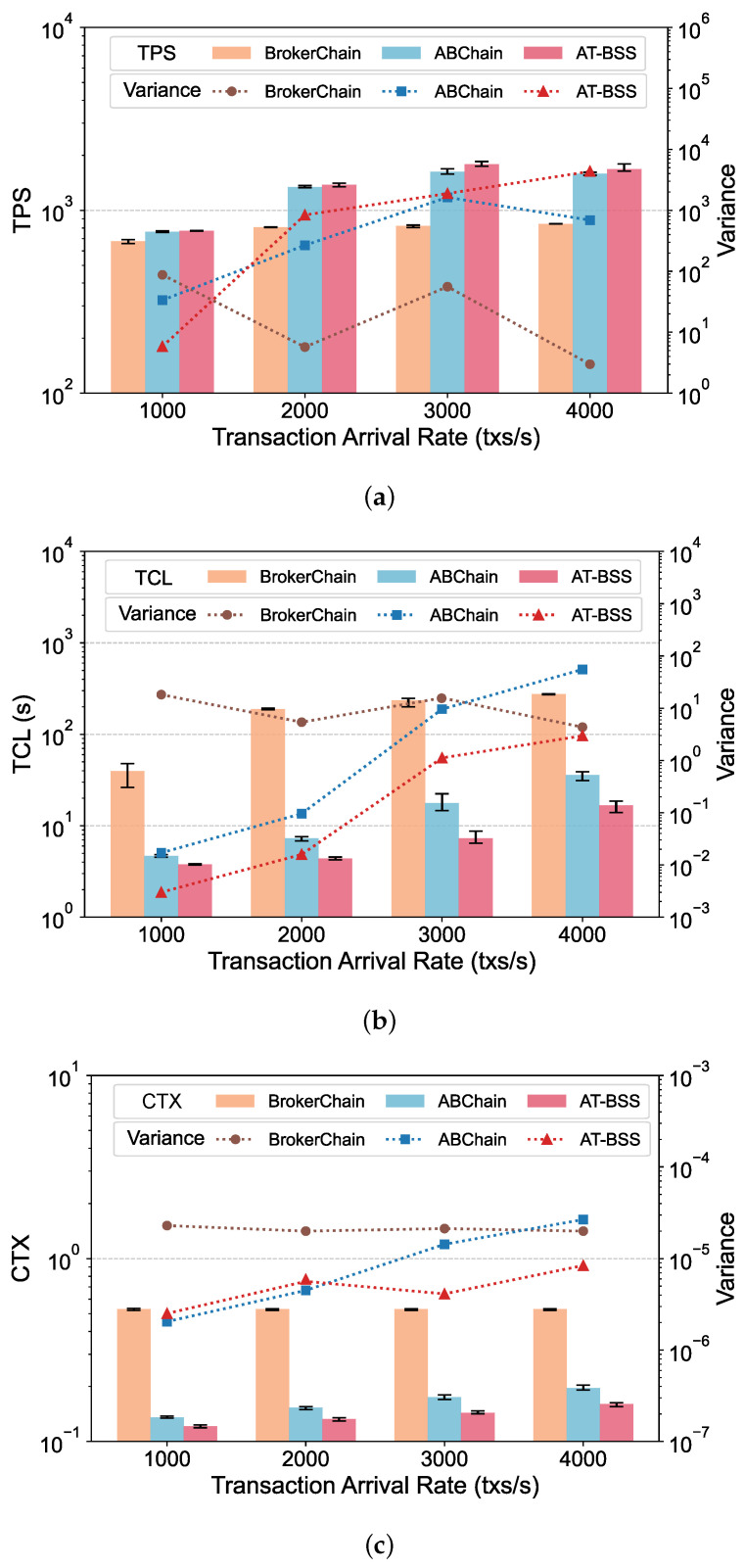
Performance comparison under varying transaction arrival rates: (**a**) TPS; (**b**) TCL; (**c**) CTX.

**Figure 7:**
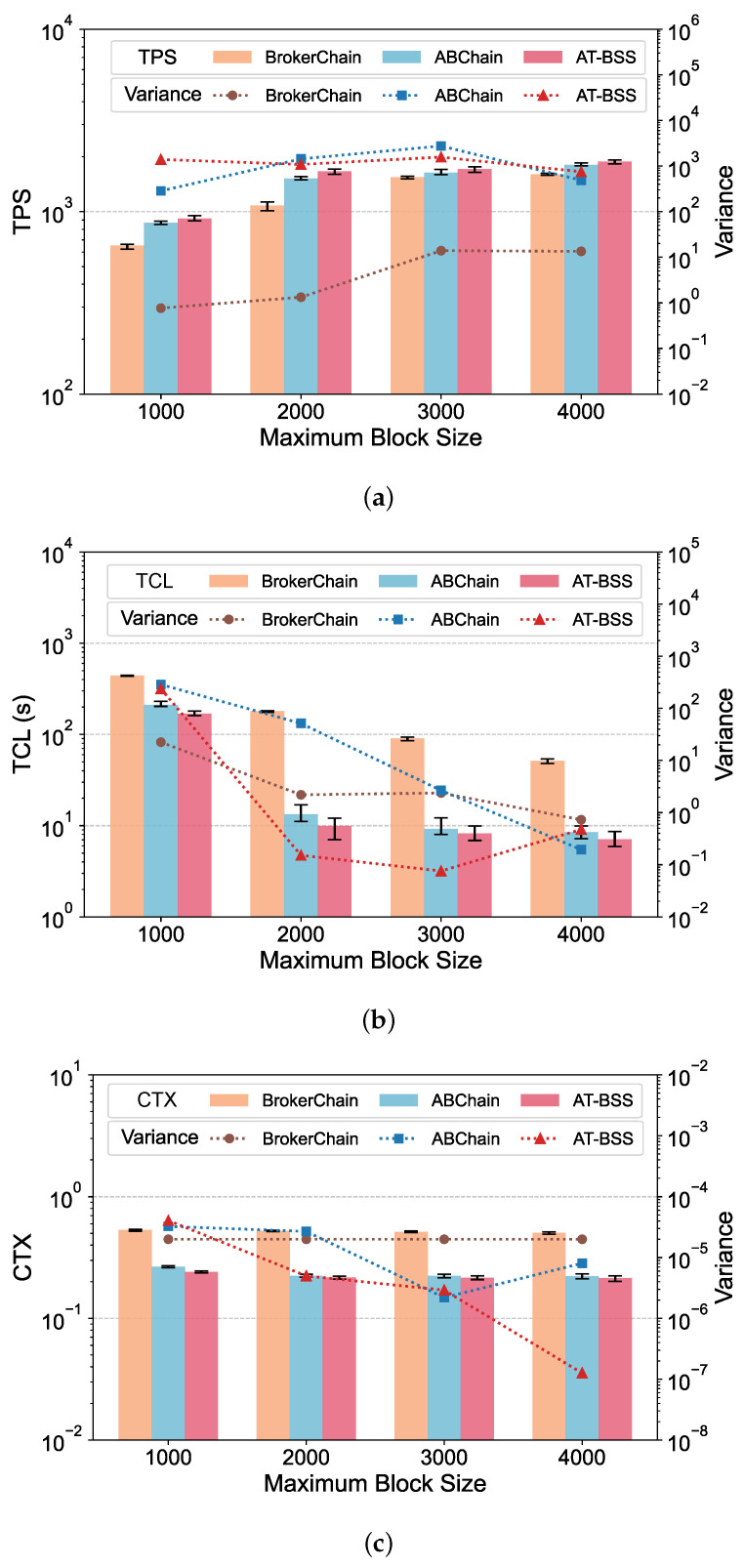
Performance comparison under varying maximum block sizes: (**a**) TPS; (**b**) TCL; (**c**) CTX.

Table 5 summarizes the main implementation and configuration details used in the experiments. Unless otherwise specified in the relevant subsections, the default system parameters are maintained as defined in Table 6. The system performance is evaluated using three core metrics: TPS, TCL, and CTX. TPS is a standard throughput metric indicating how many transactions are processed by the network each second. TCL represents transaction confirmation latency, measured from the moment a transaction is issued until it is committed to the blockchain. CTX denotes the cross-shard transaction ratio. It is computed as the number of transactions whose sender and receiver are located in different shards, divided by the total number of processed transactions. All reported results in Figure 4, Figure 5, Figure 6 and Figure 7 represent the average of five independent experimental runs. The error bars indicate the minimum-to-maximum range across these runs, and the variance is additionally reported to provide a more detailed view of the result variability. For better visualization, the values shown in the result figures are plotted on a logarithmic scale. This section displays the results on Dataset 1, while results on Dataset 2 are provided in Appendix A.

### 5.2. Impact of Broker Number

This section investigates the impact of different BrokerNum on the system performance. We varied BrokerNum in {5, 10, 15, 20, 25, 30}. As shown in Figure 4, AT-BSS consistently outperforms the baselines under all tested BrokerNum settings. Specifically, compared with ABChain, AT-BSS achieves a TPS improvement of 3.10–11.3% while reducing TCL by 50.1–60.5% and CTX by 18.1–28.7%. Compared with BrokerChain, AT-BSS delivers substantially larger improvements, boosting TPS by 106–125% and reducing TCL and CTX by 97.1–97.6% and 73.3–80.5%, respectively. These results suggest that AT-BSS not only improves throughput but also significantly mitigates cross-shard overhead, and the improvements remain as BrokerNum varies.

When BrokerNum varies, performance depends not only on how many Brokers are used but also on their workload relevance and activity. Low-relevance or inactive Brokers provide little intra-shard aggregation and can add relay and cross-shard coordination overhead, reducing throughput and increasing latency. ABChain rotates Brokers periodically, but it selects them from a fixed pool of 100 predefined accounts. As a result, the selected Brokers may not match the current workload well, and the rotation process itself introduces additional maintenance overhead. In contrast, AT-BSS selects a set of high-value Brokers from all accounts under the current system configuration. The selection is based on each account’s Accumulative Activity and Temporal Liveness. This improves the match between Brokers and transactions and reduces the probability of selecting inefficient Brokers. As a result, more transactions can be aggregated within shards, which reduces the need for additional relay transactions and lowers cross-shard message round-trip overhead.

### 5.3. Impact of Shard Number

In this section, we evaluated the impact of ShardNum variations on system performance, while keeping all other parameters at their default values. We compared AT-BSS, BrokerChain, and ABChain with ShardNum set to {2, 4, 8, 16}. The results are shown in Figure 5. As ShardNum varies, the throughput of both ABChain and BrokerChain peaks at ShardNum = 8. Moreover, AT-BSS consistently outperforms ABChain and BrokerChain across all configurations. Compared with ABChain, AT-BSS increases TPS by 2.53–4.99% and reduces TCL by 8.15–61.3%. It also lowers CTX in every setting, achieving an average CTX of 0.14–0.19, which corresponds to a 13.7–24.8% reduction. In contrast, relative to BrokerChain, AT-BSS delivers much better results. TPS improves by 7.03–229%, while TCL and CTX decrease by 9.44–97.1% and 42.3–74.3%, respectively.

As ShardNum increases, the system can process more transactions in parallel. However, partitioning the state into more shards also increases the likelihood that transactions will span multiple shards, which introduces additional cross-shard messaging, verification, and coordination delay. Under different ShardNum settings, AT-BSS selects Brokers with high activity and strong Temporal Liveness, enabling them to relay more transactions and remain effective over time. In contrast, ABChain and BrokerChain may select Brokers that are not strongly involved in transactions or are not persistently active. As a result, many transactions cannot be effectively relayed through Brokers, and the cross-shard ratio remains higher than in AT-BSS. Moreover, ABChain benefits from an adaptive Broker selection mechanism, making it more resilient than BrokerChain when the transaction changes.

### 5.4. Impact of Transaction Arrival Rate

This section presents a comparison under different transaction arrival rates. More specifically, we varied InjectSpeed to 1000, 2000, 3000, and 4000 transactions per second while keeping other parameters at default settings. The evaluation results are shown in Figure 6. As the arrival rate increases, the system workload increases, and ABChain’s TCL and CTX rise significantly. In contrast, AT-BSS is more effective at reducing cross-shard ratios and waiting overhead under high load; thus, its advantage is more pronounced at high arrival rates. Specifically, across the four arrival rates, AT-BSS consistently improves throughput and reduces both confirmation latency and cross-shard interactions. Relative to ABChain, AT-BSS increases TPS by 1.32–10.3%, with the largest result observed at 3000 tx/s, and reduces TCL by 19.4–58.8%. It also lowers CTX by 10.4–18.3%, indicating sustained suppression of cross-shard execution as load varies. Compared with BrokerChain, the advantages are more significant. TPS improves by 13.9–118%, while TCL and CTX are reduced by 90.5–97.7% and 69.8–77.0%, respectively.

As the transaction arrival rate increases, the transaction pool starts to build up and transactions spend more time waiting in the queue. In this situation, cross-shard transaction processing becomes even slower because it requires multiple rounds of cross-shard messaging and validation, which adds extra waiting on top of the queuing time. AT-BSS mitigates this effect by selecting active and strong Temporal Liveness Brokers, so more transactions can be aggregated and executed within a single shard. As a result, AT-BSS slows the growth of confirmation latency under high load.

### 5.5. Impact of Maximum Block Size

We evaluate the impact of changes in the maximum block size (MaxBlockSize) on system performance in this section. With other parameters set to their default values, the maximum block size was set to 1000, 2000, 3000, and 4000. The results are shown in Figure 7. As the maximum block size increases, the number of transactions that can be accommodated and confirmed in a single block increases, alleviating queuing pressure on the system. Therefore, throughput is more easily improved, and confirmation latency is more easily reduced. Across all four MaxBlockSize settings, AT-BSS achieves higher throughput and lower latency and cross-shard overhead than both baselines. Relative to ABChain, AT-BSS improves TPS by 1.68–15.5%, with the peak throughput observed at MaxBlockSize = 2000, while reducing TCL by 19.5–80.2% and CTX by 15.5–26.2%. The advantage over BrokerChain is more pronounced. TPS increases by 35.9–118%, and TCL and CTX decrease by 63.3–97.2% and 61.8–73.3%, respectively. The results indicate that AT-BSS remains effective under different block capacities.

Increasing the maximum block size raises the per-block capacity, so more pending transactions can be included in each block. This reduces the backlog in the transaction pool and shortens the queuing delay (i.e., the time a transaction waits before being packaged into a block). However, cross-shard transactions still incur additional coordination delay due to extra cross-shard messaging and verification, which is not eliminated by a larger block capacity. AT-BSS mitigates this and lowers the cross-shard ratio, so more transactions are executed within a single shard and cross-shard coordination overhead is avoided.

## 6. Conclusions and Future Work

Sharding technology enhances the scalability of IoT–blockchain systems through parallel processing. In this work, we identified that the inefficiency of existing Broker-based solutions in handling cross-shard transactions stems from predefined Broker selection. To bridge this gap, we proposed **AT-BSS**, a Broker selection strategy that optimizes the construction of the Broker set using two metrics: *Accumulative Activity* and *Temporal Liveness*. Using the proposed scoring metric, AT-BSS selects the top-*K* accounts with the highest scores, capturing both high transaction activity and strong temporal consistency. Our experimental evaluation on the *BlockEmulator* platform confirms the effectiveness of this approach. Across all experiments (varying BrokerNum, ShardNum, transaction arrival rate, and MaxBlockSize), AT-BSS consistently delivers higher throughput and lower confirmation latency and cross-shard ratio than both state-of-the-art baselines (ABChain and BrokerChain). Against ABChain, AT-BSS improves TPS by at least 1.32% (up to 15.5%), reduces TCL by at least 8.15% (up to 80.2%), and lowers CTX by at least 10.4% (up to 28.7%). Against BrokerChain, TPS increases by at least 7.03% (up to 229%), while TCL and CTX decrease by at least 9.44% and 42.3% (up to 97.7% and 80.5%), respectively. These results provide empirical evidence that the proposed Broker selection strategy improves scalability in sharded IoT–blockchain systems. In future work, weighted or learned combinations of features in the AT-BSS score function will be explored. In addition, we plan to evaluate AT-BSS beyond the current controlled platform in more realistic deployment environments. This will allow us to assess its robustness under real-world network conditions, such as variable latency, jitter, and packet loss. Furthermore, we plan to validate AT-BSS on IoT-native workloads, using real or synthetic device-oriented transactions. Such workloads are typically characterized by periodic communication patterns, smaller transaction values, stronger spatial locality, and event-driven burstiness. This will help determine whether the proposed Broker selection strategy remains effective under workload patterns closer to those in practical IoT deployments. Moreover, we will incorporate security-aware Broker selection mechanisms to improve robustness against activity manipulation, collusion, and targeted inactivity, thereby enhancing the reliability of AT-BSS in adversarial environments.

## Figures and Tables

**Table 1:** Comparative summary of related work.

Paper	Sharding	Main Method to Reduce Cross-Shard Transactions	Broker-Related Proposal	Application
OptChain [22]	Transaction sharding	Smart transaction placement	N/A	UTXO-based ledger
Estuary [23]	State sharding	State splitting/aggregation + COPRAS state placement	N/A	General ledger
ControlShard [24]	State sharding	Dedicated control shard	N/A	Consortium blockchain
BrokerChain [10]	State sharding	Broker accounts	Broker mechanism	Account/Balance-based ledger
BrokerFi [29]	State sharding	Broker accounts	Broker admission via staking or pledge	DeFi
Broker2Earn [30]	State sharding	Broker accounts	Incentive mechanism for Brokers	DeFi
ABChain [12]	State sharding	Broker accounts	Adaptive Brokers (periodic update)	IoT
This work	State sharding	Broker accounts	Broker selection strategy (Accumulative Activity + Temporal Liveness)	IoT

**Table 2:** Information of the full dataset source.

Aspect	Detail
Data collection	Collected by running Ethereum full nodes (from No.0 to No.23,749,999 block, until 7 November 2025)
Contents	Block, BlockTransaction, ContractInfo, etc.
Block	Contains information on 23,750,000 blocks
BlockTransaction	Contains 3,077,026,269 transactions generated from block data
ContractInfo	Contains 84,783,831 smart contracts created by 1,070,533 addresses
Storage format for BlockTransaction	Partitioned more than 20 separate files
Main fields	Block height, timestamps, sender address, receiver address, transfer amount, etc.

**Table 3:** Information of the Ethereum sub-dataset used in this study.

Dataset	Block Range	Time Span	Number of Accounts
Dataset 1	2,000,000–2,999,999	From 2016-08-02 21:32:58 UTC to 2017-01-15 10:10:14 UTC	415,735
Dataset 2	3,000,000–3,999,999	From 2017-01-15 10:10:35 UTC to 2017-07-09 20:52:20 UTC	2,855,197

**Table 4:** Three-aspect comparison of Broker selection in BrokerChain, ABChain, and AT-BSS.

Aspect	BrokerChain	ABChain	AT-BSS
Broker construction	Built-in fixed Broker list	Built-in fixed Broker list	Ranked candidate accounts
Selection principle	Predefined list	Predefined list with rotation	Score based on Activity and Liveness
Broker list size	100	100	Equal to BrokerNum

**Table 5:** Summary of the experiment and system settings.

Category	Details
Emulator	BlockEmulator (Version 1.0)
Consensus	Built-in BlockEmulator PBFT ViewChangeTimeOut: 20 s Number of consensus nodes per shard: equal to NodesInShard
Block packaging	Transactions stored in a queue Insertion: Tail append Packaging threshold: MaxBlockSize
Transaction injection	Transaction source: Ethereum historical transaction dataset Read a batch of transactions (BatchSize: 20,000) Destination of injected transactions: Assigned target shard
Congestion handling	Transactions remain queued until inclusion and no dropping
Cross-shard communication	Reliable delivery without message loss or retransmission No network delay model Cross-shard message types: Broker1Txs and Broker2Txs, representing the 1st and 2nd Broker-processing stages, respectively

**Table 6:** Initial configuration of Broker-based system parameters.

Parameter	Description	Value
NodesInShard	Number of nodes per shard	4
ShardNum	Number of shards	8
Block_Interval	Block generation interval	5 s
MaxBlockSize	Maximum block size	2000 txs/block
InjectSpeed	Transaction arrival rate	3000 txs/s
TotalDataSize	Total workload size	1,000,000 txs

**Figure A1:**
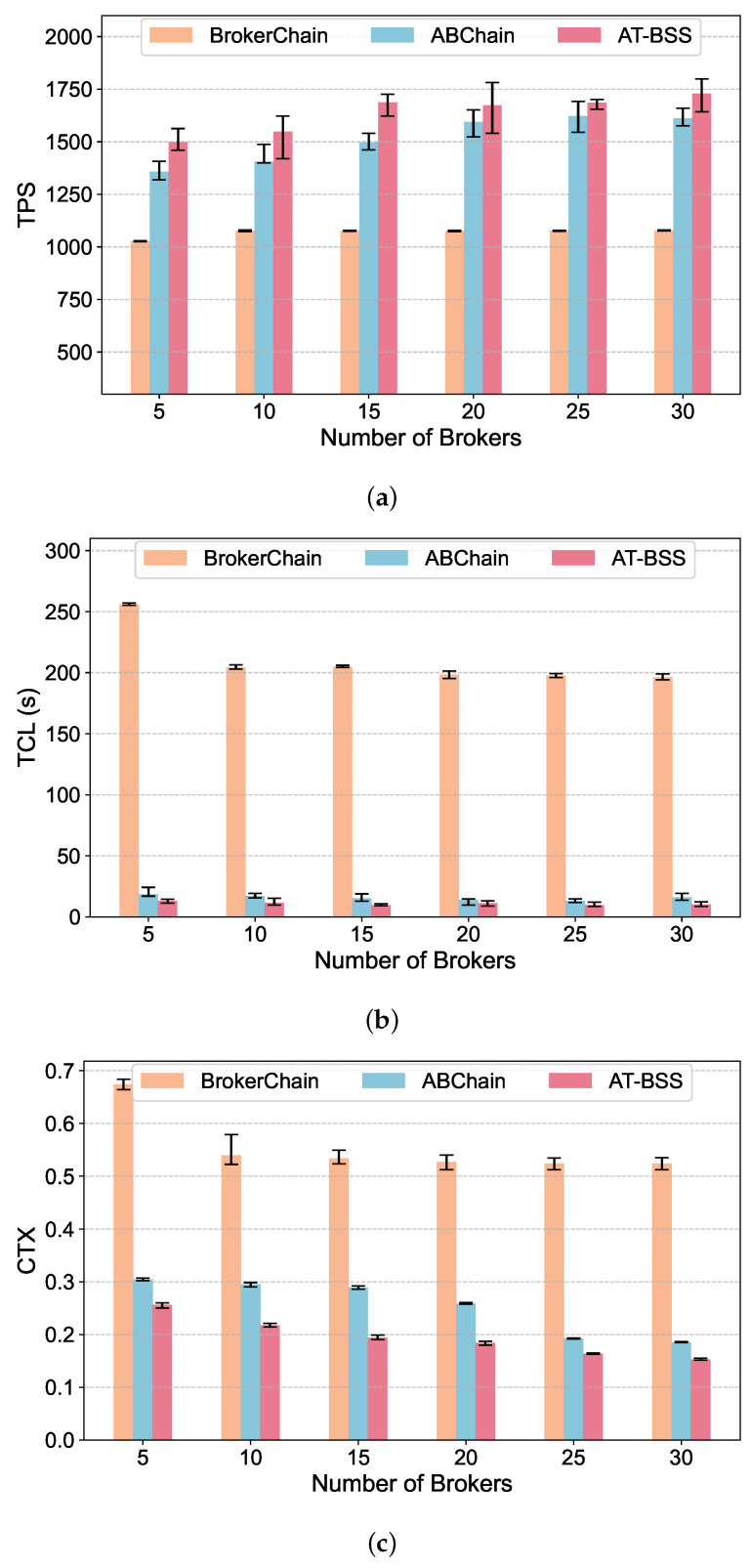
Performance comparison on Dataset 2 under varying Broker numbers: (**a**) TPS; (**b**) TCL; (**c**) CTX.

**Figure A2:**
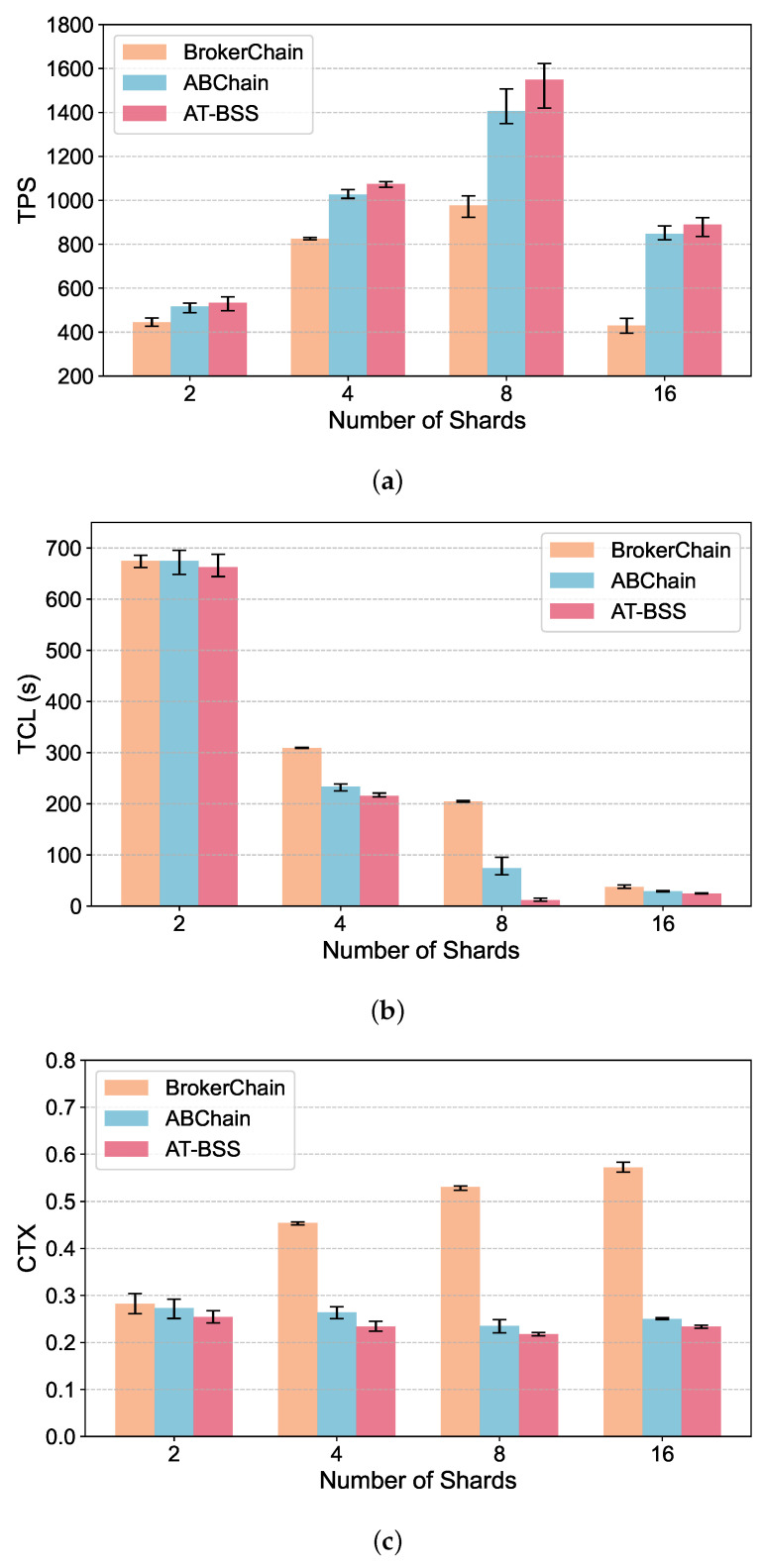
Performance comparison on Dataset 2 under varying shard numbers: (**a**) TPS; (**b**) TCL; (**c**) CTX.

**Figure A3:**
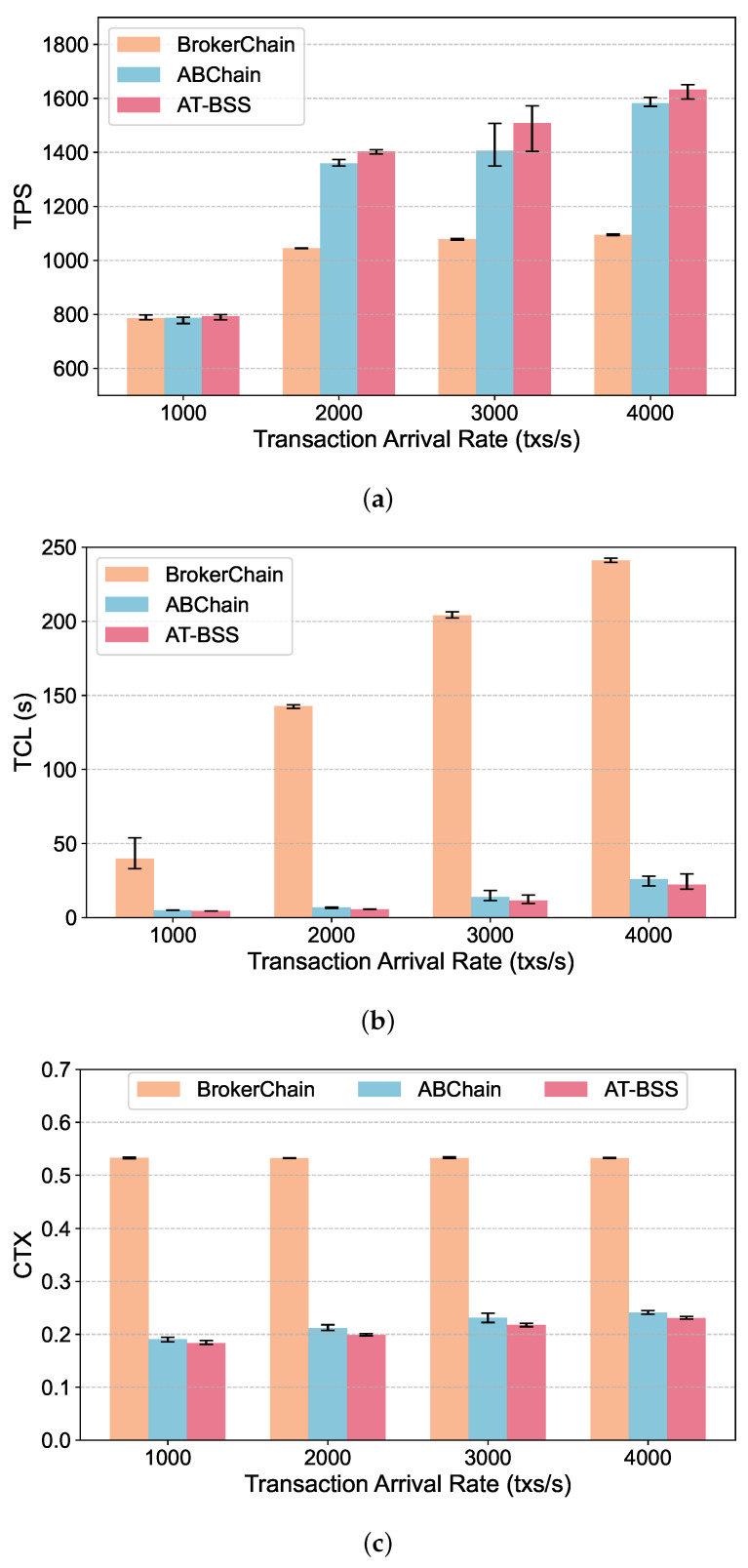
Performance comparison on Dataset 2 under varying transaction arrival rates: (**a**) TPS; (**b**) TCL; (**c**) CTX.

**Figure A4:**
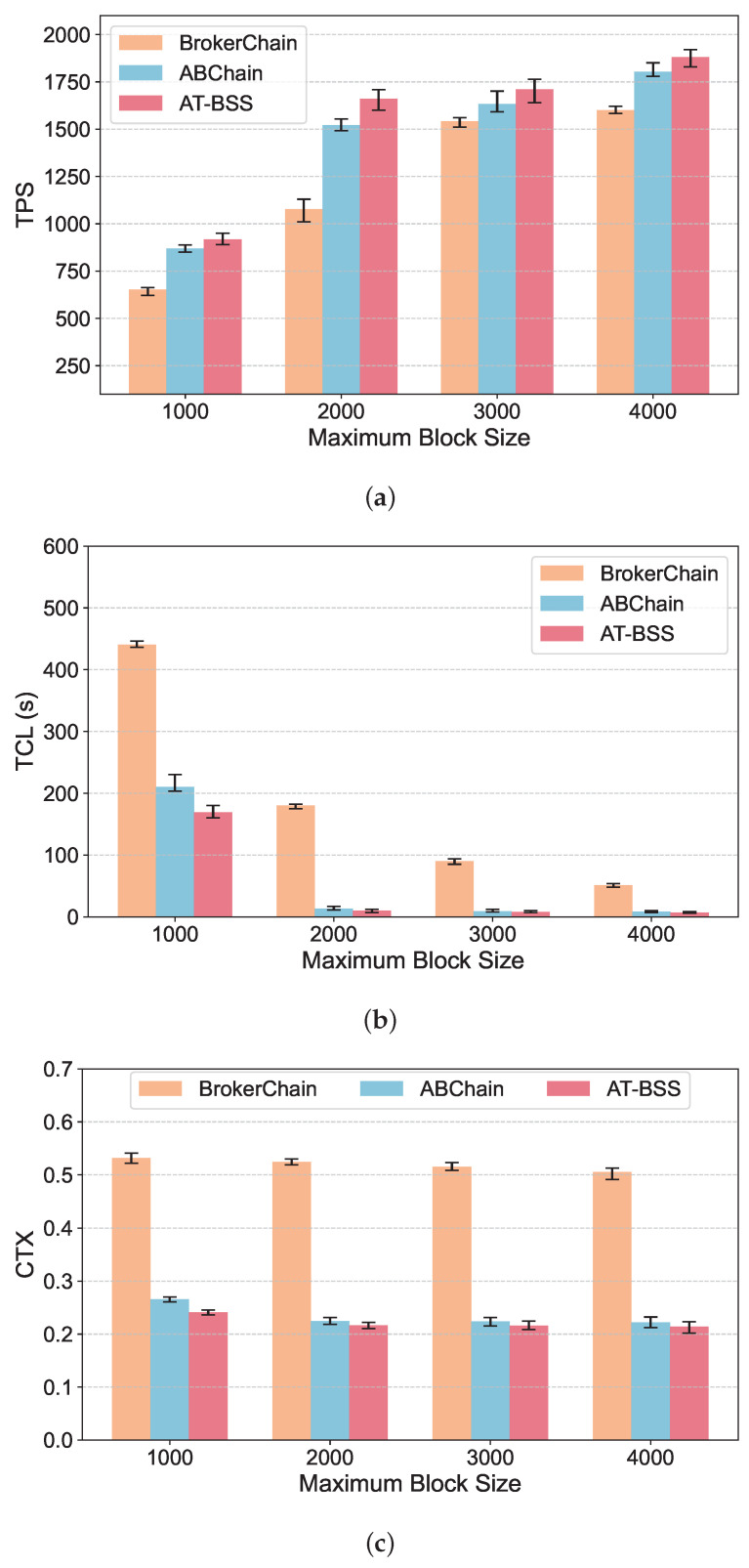
Performance comparison on Dataset 2 under varying maximum block sizes: (**a**) TPS; (**b**) TCL; (**c**) CTX.

## Data Availability

The raw data supporting the conclusions of this article will be made available by the authors on request.

## Appendix A. Experiment Results of Dataset 2

To verify that the proposed AT-BSS has better performance advantages under heterogeneous hardware environments and different datasets, we further evaluate it on a second device using Dataset 2. We compare AT-BSS with the baseline methods in terms of TPS, TCL, and CTX. The device is equipped with an AMD Ryzen 7 5700X (8-core, 3.4 GHz) CPU and 16 GB RAM, and it runs Windows 11.

The experimental results in this appendix under varying system parameters are shown in Figure A1, Figure A2, Figure A3 and Figure A4. These figures correspond to different settings of Broker number, shard number, transaction arrival rate, and maximum block size, respectively. Across all four evaluations, AT-BSS consistently achieves the highest TPS and the lowest TCL and CTX compared with ABChain and BrokerChain. The observed trends are consistent with those on dataset 1, indicating that the proposed AT-BSS strategy exhibits good generality and robustness across different system configurations and datasets.

## Appendix B. Robustness Analysis with an Alternative Temporal Liveness Descriptor

As mentioned, Temporal Liveness is characterized by the mean inter-transaction interval of an account. Although this descriptor reflects the average activity frequency, it may not fully capture bursty transaction behaviors. To examine whether the effectiveness of AT-BSS depends heavily on this specific definition, we further conduct a robustness analysis by replacing the mean-based descriptor with the variance of inter-transaction intervals. We define the variance of inter-transaction intervals as

(A1) $${\mathrm{Var}}_{\Delta t}\left(a\right)=\frac{1}{N(a)-1}\sum_{i=2}^{N(a)}{\left[\left(t_{e_{i}}-t_{e_{i-1}}\right)-\Delta t\left(a\right)\right]}^{2}.$$

where N(a) is the number of transactions associated with account *a*, $t_{e_{i}}$ is the timestamp of the *i*-th transaction of account *a* after sorting all its transactions in chronological order, and Δt(a) denotes the mean inter-transaction interval of account *a*.

To further examine the robustness of AT-BSS, we apply the variance-based descriptor to Broker selection and compare the resulting system performance with that obtained using the mean-based descriptor. Taking BrokerNum = 10 as an example, Table A1 shows that the mean-based descriptor achieves slightly better performance than the variance-based one. However, the overall conclusions remain consistent across the two methods, suggesting that the effectiveness of AT-BSS is not strongly tied to a specific definition of Temporal Liveness.

**Table A1:** Experimental results obtained using the mean-based and variance-based Temporal Liveness descriptors.

	Mean-Based Inter-Transaction Interval			Variance-Based Inter-Transaction Interval		
	TPS (txs/s)	TCL (s)	CTX	TPS (txs/s)	TCL (s)	CTX
Mean	1814	6.79	0.142	1766	7.11	0.148
Variance	2544	0.43	7.42 × $10^{-6}$	1813	0.31	5.73 × $10^{-6}$
Max	1871	7.77	0.145	1807	7.89	0.152
Min	1752	6.18	0.138	1720	6.64	0.146
